# Changes in GABAergic Transmission to and Intrinsic Excitability of Gonadotropin-Releasing Hormone (GnRH) Neurons during the Estrous Cycle in Mice

**DOI:** 10.1523/ENEURO.0171-18.2018

**Published:** 2018-11-08

**Authors:** Caroline Adams, Xi Chen, Suzanne M. Moenter

**Affiliations:** 1 Department of Molecular and Integrative Physiology, University of Michigan, Ann Arbor, MI 48109; 2 Department of Internal Medicine, University of Michigan, Ann Arbor, MI 48109; 3 Department of Obstetrics and Gynecology, University of Michigan, Ann Arbor, MI 48109

**Keywords:** action potential, estradiol, excitability, feedback, GABA, GnRH

## Abstract

Gonadotropin-releasing hormone (GnRH) neurons form the final common central output pathway controlling fertility and are regulated by steroid feedback. In females, estradiol feedback action varies between negative and positive; negative feedback typically regulates episodic GnRH release whereas positive feedback initiates a surge of GnRH, and subsequently luteinizing hormone (LH) release ultimately triggering ovulation. During the estrous cycle, changes between estradiol negative and positive feedback occur with cycle stage and time of day, with positive feedback in the late afternoon of proestrus in nocturnal species. To test the hypotheses that synaptic and intrinsic properties of GnRH neurons are regulated by cycle stage and time of day, we performed whole-cell patch-clamp studies of GnRH neurons in brain slices from mice at two times considered negative feedback (diestrous PM and proestrous AM) and during positive feedback (proestrous PM). GABAergic transmission can excite GnRH neurons and was higher in cells from proestrous PM mice than cells from proestrous AM mice and approached traditional significance levels relative to cells from diestrous PM mice. Action potential response to current injection was also greater in cells from proestrous PM mice than the other two groups. Interestingly, the hormonal milieu of proestrous AM provided stronger negative feedback on both GnRH neuron excitability and GABAergic postsynaptic current (PSC) amplitude than diestrous PM. These observations demonstrate elements of both synaptic and intrinsic properties of GnRH neurons are regulated in a cycle-dependent manner and provide insight into the neurobiological mechanisms underlying cyclic changes in neuroendocrine function among states of estradiol negative and positive feedback.

## Significance Statement

Infertility affects 15–20% of couples; failure to ovulate is a common cause. Understanding how the brain controls ovulation is critical for new developments in both infertility treatment and contraception. Gonadotropin-releasing hormone (GnRH) neurons are the final output pathway for central control of fertility and produce a signal that ultimately initiates ovulation in response to estradiol positive feedback. We studied how the reproductive cycle regulates both synaptic transmission to GnRH neurons and excitability of these cells. Both GABAergic transmission to GnRH neurons and GnRH neuron excitability are decreased during stages of the estrous cycle characterized by negative feedback by gonadal steroids, compared to the late afternoon of proestrus, when positive feedback and ovulation occur.

## Introduction

Gonadotropin-releasing hormone (GnRH) neurons form the final hormonal output pathway through which the central nervous system controls reproduction. GnRH triggers the release of the anterior pituitary hormones, LH and follicle-stimulating hormone (Schally et al., 1971), which in turn promote sex steroid production and gametogenesis. In both sexes, gonadal steroid feedback controls GnRH release and pituitary response to GnRH (Levine and Ramirez, 1982; Karsch et al., 1987; Moenter et al., 1991). For most of the female reproductive cycle, the steroid estradiol suppresses total GnRH/LH release (negative feedback). However, at the end of the follicular phase (proestrus in rodents), sustained rising levels of estradiol switch from suppressing GnRH/LH release to inducing a surge of GnRH/LH release (positive feedback; Moenter et al., 1991; Czieselsky et al., 2016). The LH surge triggers ovulation.

In rodents, ovulation is tightly coupled to time-of-day (Everett and Sawyer, 1950; Sarkar et al., 1976). GnRH/LH surges typically begin ∼1–2 h before lights out in nocturnal species with this positive feedback mode of hormone release being confined to the proestrous phase of the estrous cycle. Several paradigms for inducing positive feedback “surge” hormone release have been developed to study this phenomenon, with most involving ovariectomy and estrogen replacement (Norman et al., 1973; Legan and Karsch, 1975; Bronson and Vom Saal, 1979a; Bronson, 1981; Christian et al., 2005). Most studies of the biophysical properties of GnRH neurons during estradiol negative and positive feedback have made use of estrogen replacement surge-induction models.

To lay a basis for understanding how synaptic and intrinsic properties of GnRH neurons change between conditions of estradiol negative and positive feedback within the normal estrous cycle, we examined the rates of GABAergic fast synaptic transmission, the primary fast synaptic input to GnRH neurons, as well as GnRH neuron excitability, measured as action potential firing rate in response to current injection, and action potential properties. We compared proestrous PM, the time of positive feedback, with a low estradiol negative feedback stage (diestrous PM) and a high estradiol negative feedback stage (proestrous AM). We hypothesized that the transition among cycle stages induces changes in the intrinsic properties of GnRH neurons and GABAergic fast synaptic transmission to these cells.

## Materials and Methods

All chemicals were purchased from Sigma-Aldrich, unless noted.

### Animals

Transgenic mice expressing green fluorescent protein (GFP) under the control of the GnRH promoter (Tg(Gnrh1-EGFP)51Sumo MGI:6158457; GnRH-GFP mice) were used (Suter et al., 2000). Mice were housed on a 14/10 h light/dark cycle with lights off at 6 P.M. (eastern standard time). Teklad 2916 chow (Envigo) and water were available ad libitum. Estrous cycles of adult females aged 60–128 d were monitored by vaginal cytology to determine estrous cycle stage; mice were studied on diestrus or proestrus. Uterine mass was measured at the time of brain slice preparation to confirm cycle stage as it is directly proportional to circulating estradiol levels (Shim et al., 2000). Uterine mass was within the published range for diestrus (*n* = 10, 47.3 ± 2.7 mg) and proestrus (AM, *n* = 8, 131.3 ± 10.6 mg; PM, *n* = 11, 121.5 ± 2.7 mg; Silveira et al., 2017). Uterine mass was lower on diestrus (one-way ANOVA/Tukey’s *F*_(2,26)_ = 68.3, *p* < 0.0001) than either time of day on proestrus and was not different between proestrous AM and PM (*p* > 0.4).

### Brain slice preparation

All solutions were bubbled with 95% O_2_/5% CO_2_ throughout the experiments and for at least 15 min before exposure to tissue. Brain slices for AM recordings were prepared 8.5–9.5 h before lights out; slices for PM recordings were prepared 1.5–2.5 h before lights out. The time of PM slice preparation corresponds to 30 min before the onset “surge peak” window through the end of that window as defined in previous work (Christian and Moenter, 2007). The brain was rapidly removed and placed in ice-cold sucrose saline solution containing the following: 250 mM sucrose, 3.5 mM KCl, 26 mM NaHCO_3_, 10 mM D-glucose, 1.25 mM Na_2_HPO_4_, 1.2 mM MgSO_4_, and 3.8 mM MgCl_2_, at pH 7.6 and 345 mOsm. Coronal (300 µm) slices were cut with a VT1200S Microtome (Leica Biosystems). Slices were incubated in a 1:1 mixture of sucrose saline and artificial CSF (ACSF) containing 135 mM NaCl, 3.5 mM KCl, 26 mM NaHCO_3_, 10 mM D-glucose, 1.25 mM Na_2_HPO_4_, 1.2 mM MgSO_4_, and 2.5 mM CaCl_2_, at pH 7.4 and 305 mOsm, for 30 min at room temperature (∼21–23°C). Slices were then transferred to 100% ACSF at room temperature for 0.5–5 h before recording.

### Data acquisition

During recording, slices containing the preoptic area and anterior hypothalamus, which contain the majority of GnRH neuron somata, were placed into a chamber continuously perfused with ACSF at a rate of 2 ml/min with oxygenated ACSF heated to 29.5–31.5°C with an inline-heating unit (Warner Instruments). GFP-positive cells were visualized with a combination of infrared differential interference contrast and fluorescence microscopy on an Olympus BX50WI or BX51WI microscope. Borosilicate glass capillaries (1.65-mm OD × 1.12-mm ID; World Precision Instruments, Inc.) were pulled by using a Flaming/Brown P-97 unit (Sutter Instrument Company) to make recording pipettes. Pipettes measured 2–4.5 MΩ when filled with: 125 mM K gluconate, 20 mM KCl, 10 mM HEPES, 5 mM EGTA, 0.1 mM CaCl_2_, 4 mM MgATP, and 0.4 mM NaGTP; 300 mOsm, pH 7.2 with NaOH for current-clamp recordings or when filled with: 140 mM KCl, 10 mM HEPES, 5 mM EGTA, 0.1 mM CaCl_2_, 4 mM MgATP, and 0.4 mM NaGTP; 300 mOsm, pH 7.2 with NaOH for recording GABAergic PSCs. Pipettes were wrapped with Parafilm (Bemis) to reduce capacitive transients; remaining transients were electronically cancelled. Pipettes were placed in contact with a GFP-positive neuron using an MP-285 micromanipulator (Sutter Instrument Company). All potentials reported were corrected online for liquid junction potential of −14.2 mV for the K gluconate pipette solution and 4.9 mV for the 140 mM KCl pipette solution. (Barry, 1994). Recordings were made with an EPC-10 dual patch-clamp amplifier (HEKA Elektronik) and Patchmaster software (HEKA Elektronik). Experiments were analyzed offline using custom software (DeFazio and Moenter, 2002; DeFazio et al., 2014) written in IgorPro (Wavemetrics).

### Experimental design

Comparisons of the properties of GABAergic transmission to GnRH neurons and the intrinsic firing properties of GnRH neurons in response to current injection were made among cells in brain slices from diestrous PM, proestrous AM, and proestrous PM mice.

### Whole-cell patch-clamp

After achieving a >1 GΩ seal and the whole-cell configuration, membrane potential was held at -60 mV between protocols. Series resistance (R_s_), input resistance (R_in_), and holding current (I_hold_) were measured every 2–3 min using a 5-mV hyperpolarizing step from −60 mV (mean of 20 repeats, 20-ms duration, sampled at 100 kHz and filtered at 10 kHz). Only recordings with a R_in_ of >500 MΩ, I_hold_ of −50 − 20 pA, stable R_s_ of <20 MΩ, and a stable Cm between 8.5 and 23 pF were used for analysis.

Spontaneous GABAergic postsynaptic currents (sPSCs) were measured in voltage-clamp at a holding potential of -70 mV. Current was sampled at 10 kHz and filtered at 10 kHz. ACSF contained 20 μM D-APV (Tocris), and 20 μM CNQX to block ionotropic glutamate receptors. At least two 120-s recordings were made for each cell for determining sPSC frequency. Mean ± SEM recording time was 591 ± 107 s/cell for diestrous PM (*n* = 11, range 240–1200 s), 457 ± 46 s/cell for proestrous AM (*n* = 9, range 240–600 s), and 536 ± 56 s/cell during proestrus PM (*n* = 16, range 244–1010 s). A total of 1351, 446, and 7929 sPSC events were recorded during diestrous PM, proestrous AM, and proestrous PM, respectively.

To measure activity-independent miniature PSCs (mPSCs), at least two to three 120-s recordings were made before and during bath application of 1 μM tetrodotoxin (TTX, Tocris) in a separate set of cells from the diestrous PM and proestrous PM groups.

GnRH neuron excitability was assessed in current-clamp recordings. Direct current was adjusted to keep cells within 2 mV of -69 mV. Membrane potential was sampled at 20 kHz and filtered at 7.3 kHz. Bridge balance (95%) was used for most cells; for a few cells in diestrous PM and proestrous PM groups, bridge balance was not used but results were similar. ACSF contained 100 μM picrotoxin, 20 μM D-APV, and 20 μM CNQX to block ionotropic GABA and glutamate receptors. Cells were injected with current from 0 to 30 pA (500 ms, 2-pA steps). This protocol was repeated two to three times per cell and the number of action potentials at each step was averaged. The first spike fired was used to determine the following action potential characteristics: latency from start of the current injection to first spike, firing threshold (membrane potential when the first derivative of the voltage trace exceeds 1 V/s), peak amplitude relative to threshold, full width at half-maximum (FWHM), rate-of-rise, and time and amplitude of after-hyperpolarization potential (AHP; both relative to threshold).

### Statistical analyses

Data were analyzed using Prism 7 (GraphPad) or SPSS (IBM) and are reported as the mean ± SEM. The number of cells per group is indicated by *n*. No more than two cells were used per animal with at least four animals tested per group. One cell from the diestrous PM GABA transmission group was identified as an outlier by ROUT (robust regression and outlier removal) with a strict Q coefficient of 0.01 and was excluded from all data sets. Data distribution was determined using a Shapiro–Wilk test for normality. Amplitudes of sPSC were binned at 5-pA intervals and histograms constructed of the mean on a per cell basis. Interevent intervals were binned at 0.1 s and plotted as a cumulative probability; events in cells from the proestrous AM group were sufficiently infrequent that the histogram of these data was not informative. Recordings with zero events were excluded from interevent interval analysis; values reported are thus an underestimate of interevent interval as the maximum that could be considered was two minutes. ANOVA analyses did not assume equal subgroup sizes. Tests are specified in the results and legends; *p* < 0.05 was accepted as significant.

## Results

### GABAergic transmission to GnRH neurons is increased during proestrus

**Figure 1.**
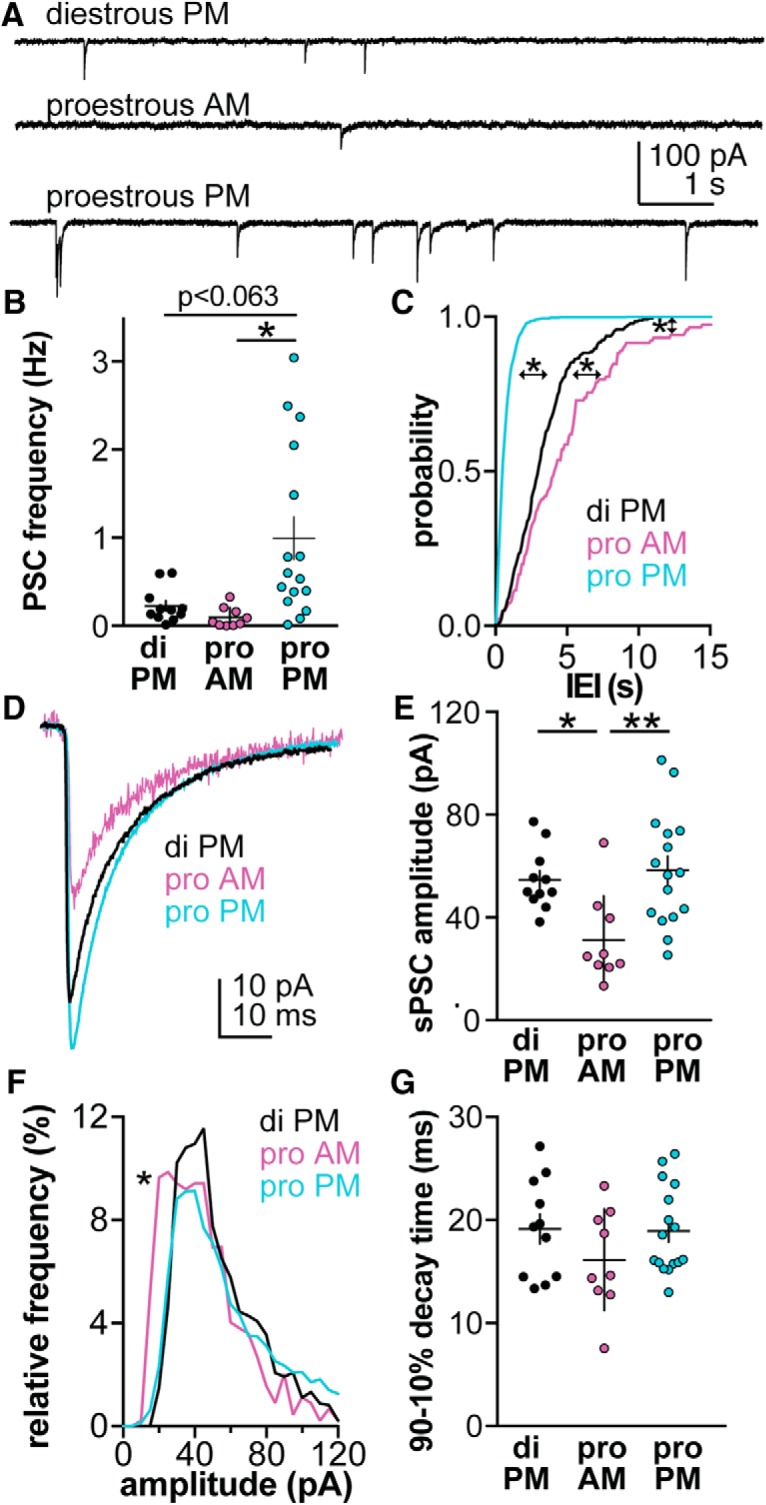
GABAergic sPSC frequency is highest on proestrous PM. ***A***, Representative sPSC recording from a neuron in each group. ***B***, Individual values and mean ± SEM of spontaneous GABAergic PSC frequency for cells recorded on diestrous (di) PM, proestrous (pro) AM and pro PM (Kruskal–Wallis, KW = 14.4, *p < 0.05 Dunn’s). ***C***, Mean by-cell cumulative probability distribution of interevent interval (IEI) for each group (Kruskal–Wallis, KW = 191, *p < 0.0001, Dunn’s). ***D***, By-cell average of all sPSC from all cells in each group. ***E***, Individual values and mean ± SEM of sPSC amplitude (ANOVA *F*_(2,33)_ = 6.69, *p < 0.05, **p < 0.005 Tukey). ***F***, Histogram of mean by-cell sPSC amplitude distribution (Kruskal–Wallis, KW = 23.9, proestrous AM vs both diestrous PM and proestrous PM, *p < 0.001, Dunn’s). ***G***, Individual values and mean ± SEM of sPSC time decay time between 90% and 10% of the maximum event amplitude (ANOVA *F*_(2,33)_ = 1.34).

In the daily surge model (Christian et al, 2005), GABAergic transmission is decreased during negative feedback and increased during positive feedback relative to OVX controls (Christian and Moenter, 2007). To examine whether GABA transmission to GnRH neurons is modulated between phases of the estrous cycle during which physiologic negative versus positive feedback are observed, GABAergic sPSCs were recorded from GnRH neurons in brain slices obtained from diestrous PM, proestrous AM (both negative feedback), or proestrous PM (positive feedback) mice. Representative recordings are shown in Figure 1*A*, and recording parameters in Table 1. Frequency of GABAergic sPSCs was increased during proestrous PM relative to proestrous AM and approached traditional significance values versus diestrous PM (Fig. 1*B*; diestrous PM *n* = 11, proestrous AM *n* = 9, proestrous PM *n* = 16, Kruskal–Wallis/Dunn’s, *p* = 0.063 proestrous PM vs diestrous PM, *p* < 0.001 proestrous AM vs proestrous PM). Interestingly, although mean frequency of GABA transmission from cells recorded on diestrous PM was not different from proestrous AM, the cumulative probability distribution of sPSC interevent interval averaged by cell differed significantly among all groups. Specifically, the distribution was shifted toward shorter intervals on proestrous PM and longer intervals for proestrous AM, both being different from the intermediate distribution for diestrous PM events and from one another (Fig. 1*C*; Kruskal–Wallis/Dunn’s, proestrous AM vs both proestrous PM and diestrous PM, *p* < 0.0001; diestrous PM vs proestrous PM, *p* < 0.0001). Cumulative distributions can be skewed by one or two high frequency cells; in these data sets, the median and interquartile range followed the same pattern as the mean (diestrous PM 0.18 Hz [IQR 0.10–0.32], proestrous AM 0.02 Hz [0.001–0.16], proestrous PM 0.57 Hz [0.30–1.91]).

**Table 1:** **. Whole-cell recording properties for** Figures 1–3

Mean ± SEM of GnRH whole-cell passive properties from Figure 1				
	Diestrous PM	Proestrus AM		Proestrus PM
R_in_ (MΩ)	929 ± 42	1336 ± 160*		1034 ± 63
Capacitance (pF)	14.7 ± 0.7	13.0 ± 0.8		14.5 ± 0.7
R_s_ (MΩ)	13.2 ± 0.6	13.5 ± 1.0		14.6 ± 0.7
I_hold_ (pA)	-13.6 ± 4.2	-0.15 ± 4.7^#^		-17.4 ± 2.8
*p < 0.05 vs diestrous PM; #*p* < 0.01 vs proestrous PM, Tukey’s				
ANOVA parameters for comparison of GnRH passive properties (Fig. 1)				
R_in_ (MΩ)	*F*_(2,33)_ = 4.84			
Capacitance (pF)	*F*_(2,33)_ = 1.32			
R_s_ (MΩ)	*F*_(2,33)_ = 0.951			
I_hold_ (pA)	*F*_(2,33)_ = 5.38			
Mean ± SEM of GnRH whole-cell passive properties from Figure 2				
	Diestrous PM	Proestrus PM		
R_in_ (MΩ) Before TTX During TTX	1053 ± 88 846 ± 83	1113 ± 145 775 ± 91		
Capacitance (pF) Before TTX During TTX	15.8 ± 0.8 15.0 ± 0.8	13.2 ± 0.9 13.8 ± 1.0		
R_s_ (MΩ) Before TTX During TTX	12.1 ± 0.9 13.3 ± 1.5	12.3 ± 0.6 14.7 ± 0.8		
I_hold_ (pA) Before TTX During TTX	-16.4 ± 3.5 -25.1 ± 6.3	-19.4 ± 2.6 -28.5 ± 3.8		
Two-way repeated measures ANOVA for comparison of GnRH passive properties among groups (Fig. 2)				
	Group	TTX	Group × TTX	
R_in_ (MΩ)	*F*_(1,9)_ = 0.001	*F*_(1,9)_ = 36.0***	*F*_(1,9)_ = 2.1	
Capacitance (pF)	*F*_(1,9)_ = 2.5	*F*_(1,9)_ = 0.1	*F*_(1,9)_ = 6.9*	
R_s_ (MΩ)	*F*_(1,9)_ = 0.4	*F*_(1,9)_ = 6.0*	*F*_(1,9)_ = 0.7	
I_hold_ (pA)	*F*_(1,9)_ = 0.3	*F*_(1,9)_ = 14.1**	*F*_(1,9)_ = 0.01	
Mean ± SEM of GnRH whole-cell passive properties from Figure 3				
	Diestrous PM	Proestrous AM		Proestrous PM
R_in_ (MΩ)	1125 ± 150	667 ± 43*		1361 ± 144
Capacitance (pF)	13.7 ± 0.7	13.8 ± 0.7		12.5 ± 0.8
R_s_ (MΩ)	13.5 ± 0.9	11.9 ± 0.9		13.5 ± 1.4
I_hold_ (pA)	-0.7 ± 5.2	-2.8 ± 6.3		-10.1 ± 4.4
*p < 0.05 vs diestrous PM, Tukey’s				
ANOVA parameters for comparison of GnRH passive properties (Fig. 3)				
R_in_ (MΩ)	*F*_(2,22)_ = 6.65			
Capacitance (pF)	*F*_(2,22)_ = 1.02			
R_s_ (MΩ)	*F*_(2,22)_ = 0.62			
I_hold_ (pA)	KW = 3.36			

Amplitude of sPSCs was also markedly suppressed in cells from proestrous AM mice (Fig. 1*D*,*E*; one-way ANOVA/Tukey, proestrous AM *p* < 0.05 vs diestrous PM, proestrous AM *p* < 0.005 vs proestrous PM). Consistent with this observation, the peak of the amplitude histogram was significantly left-shifted for proestrous AM cells versus diestrous PM and proestrous PM cells (Fig. 1*F*; Kruskal–Wallis/Dunn’s, *p* < 0.001). No difference was observed in decay time between 90% and 10% of the maximum current amplitude (Fig. 1*G*; ANOVA, *p* > 0.2).

### GABAergic transmission is primarily activity independent and does not change between diestrus and proestrus

**Figure 2.**
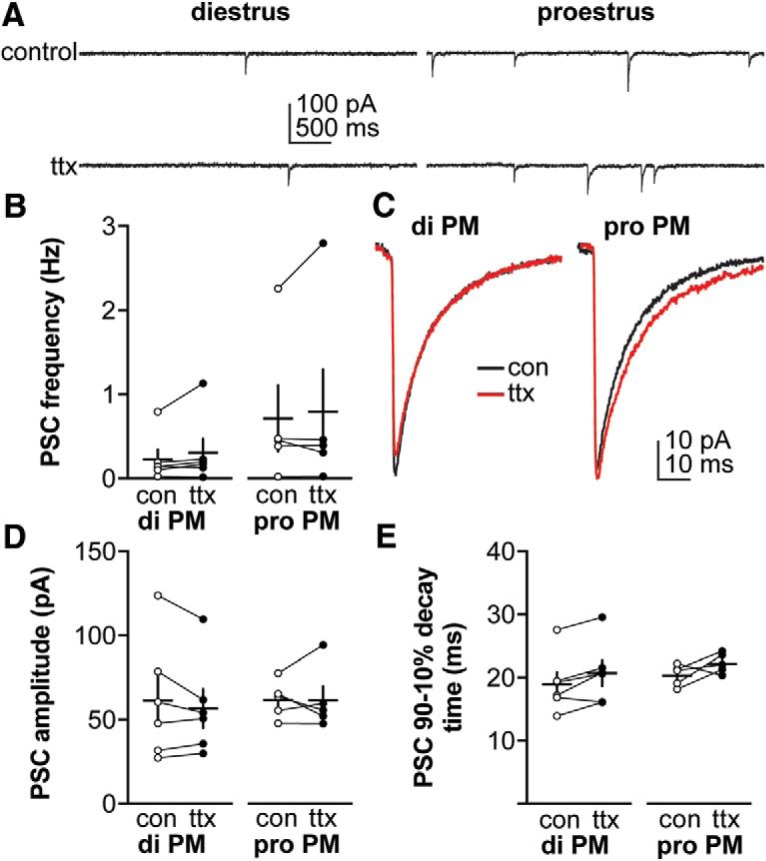
Blocking action potentials does not affect GABAergic PSC frequency or amplitude in diestrous or proestrous mice. ***A***, Representative recordings from a representative neuron in each group before (control or con, top) and during (bottom) TTX treatment (from *n* = 6 cells diestrous PM, *n* = 5 cells proestrous PM). ***B***, Individual values and mean ± SEM of GABAergic PSC frequency. ***C***, Average of all PSC traces for control or ttx periods from all cells in each group. ***D***, ***E***, Individual values and mean ± SEM for: (***D***) PSC amplitude, (***E***) decay time between 90% and 10% of the maximum current amplitude. No statistical differences were detected using two-way repeated-measures ANOVA/Bonferroni test (***B***, cycle stage: *F*_(1,9)_ = 1.3; TTX: *F*_(1,9)_ = 1.6; cycle stage × TTX: *F*_(1,9)_ = 0.0; ***D***, cycle stage: *F*_(1,9)_ = 0.3; TTX: *F*_(1,9)_ = 0.6; cycle stage × TTX: *F*_(1,9)_ = 0.5; ***E***, cycle stage: *F*_(1,9)_ = 0.5; TTX: *F*_(1,9)_ = 6.4 (*p* = 0.01); cycle stage × TTX: *F*_(1,9)_ = 0.9).

Increased GABAergic PSC frequency during proestrus may be due to an increase in presynaptic activity and/or synaptic release sites on GnRH neurons. To differentiate between these mechanisms, PSC frequency and amplitude were recorded before and during treatment with the voltage-gated sodium channel blocker TTX (Fig. 2*A*). TTX treatment isolates activity-independent neurotransmission, which is proportionate to the number of functional synaptic connections as well as to release probability at individual release sites (Auger and Marty, 2000; Kaeser and Regehr, 2014). Because the frequency of overall GABAergic transmission was very low in cells recorded on proestrous AM, they were excluded from this analysis. Neither PSC frequency nor amplitude (Fig. 2*B–D*; two-way repeated-measures ANOVA/Bonferroni) were altered during TTX treatment (*n* = 6 cells diestrous PM, *n* = 5 cells proestrous PM). An increase in PSC decay time during TTX was detected by ANOVA, but *post hoc* tests did not detect differences within cycle stage (Fig. 2*E*; two-way repeated-measures ANOVA, TTX: *F*_(1,9)_ = 6.4, Bonferroni: *p* = 0.22 for both groups). Collectively these data indicate that most synaptic transmission observed in the slice is activity independent and that this does not change between the cycle stages examined.

### GnRH neuron excitability is increased during positive feedback

**Figure 3.**
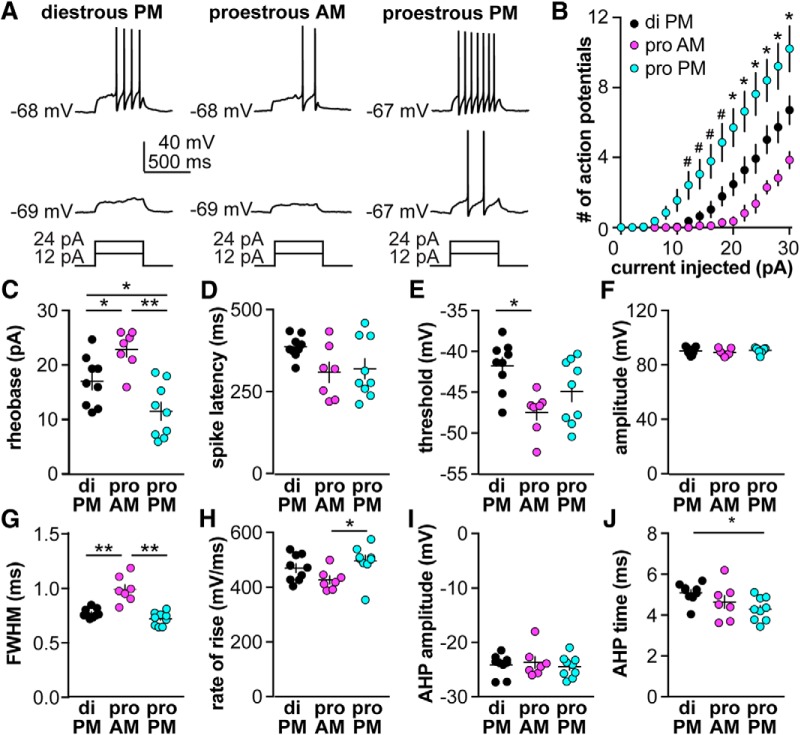
GnRH neuron excitability is increased on proestrus versus diestrus. ***A***, Representative traces from a neuron in each group during 500-ms current injections of 12 and 24 pA (current injection protocol below). ***B***, Mean ± SEM spikes elicited for each current injection step (two-way repeated-measures ANOVA cycle stage: *F*_(2,22)_ = 10.2, current: *F*_(15,330)_ = 93.03, interaction: *F*_(30,330)_ = 9.503, *p < 0.05 diestrous PM vs proestrous PM and *p* < 0.05 proestrous AM vs proestrous PM; *p < 0.05 among all three groups, Fisher’s LSD). ***C–H***, Individual values and mean ± SEM for: (***C***) rheobase current (ANOVA *F*_(2,22)_ = 12.8, *p < 0.05, **p < 0.0001), (***D***) latency to first spike (ANOVA *F*_(2,22)_ = 2.85, *p* = 0.0792), (***E***) action potential threshold (ANOVA *F*_(2,22)_ = 6.18, *p < 0.01 Tukey), (***F***) action potential amplitude (ANOVA, *F*_(2,22)_ = 0.676), (***G***) FWHM (ANOVA *F*_(2,22)_ = 26.2, **p < 0.0001 Tukey), (***H***) action potential rate of rise (Kruskal–Wallis, KW = 6.69, *p < 0.05 Dunn’s), (***I***) AHP amplitude (ANOVA *F*_(2,22)_ = 0.252), and (***J***) AHP time (Kruskal–Wallis, KW = 7.03, *p < 0.05 Dunn’s).

To investigate whether GnRH neuron excitability is also modulated during the estrous cycle, we measured GnRH neuron response to depolarizing steady-state current injections (0–30 pA, 2-pA steps, 500 ms). Figure 3*A* shows representative responses to +12 and +24 pA injections. The rheobase current (the minimum current required to initiate spikes) was lowest on proestous PM during positive feedback and highest on proestrous AM (Fig. 3*C*; diestrous PM *n* = 9, proestrous AM *n* = 7, proestrous PM *n* = 9, one-way ANOVA/Tukey, *p* < 0.05 diestrous PM vs both proestrous AM and proestrous PM, *p* < 0.0001 proestrous AM vs proestrous PM). Once firing was initiated, GnRH neurons from proestrous PM mice fired more spikes. Specifically, at current steps from 12 to 30 pA, cells recorded on proestrous PM fired more spikes than cells from either diestrous PM mice or proestrous AM mice (Fig. 3*B*; two-way repeated-measures ANOVA/Fisher’s LSD, *p* < 0.05). Differences were also observed between the two negative feedback stages examined; at current steps ≥20 pA, cells from proestrous AM mice fired fewer spikes than cells from diestrous PM mice. Rin was lower in cells recorded on proestrous AM; this could contribute to fewer spikes being fired in this group (Table 1).

A number of action potential properties were also altered among the cycle stages examined, including action potential threshold being hyperpolarized on proestrous AM versus diestrous PM (Fig. 3*E*; ANOVA/Tukey, *p* < 0.01) and rate-of-rise being lower on proestrous AM than proestrous PM (Fig. 3*H*; Kruskal–Wallis/Dunn’s, *p* < 0.05). FWHM was greater on proestrous AM than both other groups (Fig. 3*G*; ANOVA/Tukey, *p* < 0.0001). Afterhyperpolarization time was reduced on proestrous PM compared to diestrous PM (Fig. 3*J*; Kruskal–Wallis/Dunn’s, *p* < 0.05). No cycle-dependent changes were observed in time to first spike (spike latency; Fig. 3*D*; ANOVA *p* = 0.0792), AP spike amplitude (Fig. 3*F*; ANOVA, *p* > 0.5), or AHP amplitude (Fig. 3*I*; ANOVA, *p* > 0.5).

## Discussion

The female reproductive cycle is characterized by one of the rare examples of positive feedback in physiology, specifically the induction of a surge mode of GnRH and LH release at the end of the follicular phase (proestrous PM in nocturnal rodents). This is largely attributed to exposure to high sustained levels of estradiol from the mature Graafian follicle(s) (Docke and Dorner, 1965). Here, we show that GABAergic transmission to GnRH neurons and GnRH neuron excitability are both increased during positive feedback (proestrous PM) relative to two different representations of negative feedback, diestrous PM and proestrous AM, which, interestingly, also diverged from one another in some aspects.

The present observations support and extend work in an OVX+E mouse model in which the switch from estradiol negative to positive feedback occurs solely on a time-of-day basis, referred to as the daily surge model (Christian et al., 2005). Estradiol must be elevated near peak follicular phase levels for several hours to initiate the switch to positive feedback (Evans et al., 1997). The levels of estradiol achieved in the daily surge model (Christian et al., 2005) are in the physiologic range, but are persistently, rather than cyclically, elevated, and the result is daily changes from negative to positive feedback. Because estradiol level is similar during negative and positive feedback, it is not an estradiol rise that triggers the change between states in this model. This differs from the estrous cycle in which an estradiol rise is viewed as the trigger for the transition. The question is thus raised of whether or not underlying mechanistic differences observed between feedback states in the daily surge model are the same as those during the cycle. The similar increase in GnRH neuron excitability and GABA transmission observed during positive feedback in the present work in cycling mice to published observations in the OVX+E daily surge model indicates at least some of the neurobiological mechanisms underlying the feedback switch are similar between these models. Consistent with these observations, GnRH neuron firing rate has also been shown to be similar during positive feedback whether induced by OVX+E or occurring spontaneously on the afternoon of proestrus (Silveira et al., 2017).

Of interest, the two negative feedback stages studied also diverged from one another with regard to some of the parameters examined. Specifically, cells studied on the morning of proestrus were less excitable and had smaller amplitude GABAergic PSCs than cells on the afternoon of diestrus. Both estradiol and progesterone change with cycle stage and either or both may underlie these observations. Progesterone typically provides negative feedback on GnRH release and firing rate (Moenter et al., 1991; Barrell et al., 1992; Pielecka et al., 2006). In the present studies we have no measure of progesterone, but it is likely that the influence of this steroid would be greater on diestrous PM than proestrous AM. Based on uterine mass, we can surmise that estradiol levels were higher on the morning of proestrus than on the afternoon of diestrus. The reduced excitability and transmission observed on proestrous AM relative to diestrous PM may thus be a consequence of the increase in estradiol inducing a biphasic feedback response, with negative feedback preceding positive feedback. In this regard, administration of a surge-inducing dose of estradiol to ovariectomized females initially suppresses and then increases GnRH release (Moenter et al., 1990). Similar observations have been made during reproductive cycles of sheep and women, in which the amplitude of LH pulses is reduced as the follicular phase proceeds, coincident with rising estradiol levels. In the present study, some action potential parameters appeared to change sooner on exposure to the cyclical rise in estradiol (e.g., the hyperpolarization of threshold) than others (e.g., increased rate of rise). Still others exhibited biphasic changes on exposure to the estradiol rise (e.g., rheobase and FWHM). Together, these observations suggest both that estradiol action during the mouse cycle is biphasic and that the negative feedback signal provided by high estradiol before transition to positive feedback on proestrus is stronger than that produced by the hormonal milieu on diestrus. This indicates the strong negative feedback observed in the AM of the OVX+E daily surge model may more closely resemble proestrous AM than diestrous PM.

The shift from negative to positive feedback from proestrous AM to proestrous PM is consistent with the biphasic effects of estradiol, but may be augmented by other steroid changes. In addition to its role during negative feedback, progesterone can also amplify the LH surge in rats and mice (Bronson and Vom Saal, 1979b). Studies have also identified central changes induced by progesterone and ligand-independent actions of the progesterone receptor as important for positive feedback (Chappell et al., 1999; Chappell and Levine, 2000; Micevych and Sinchak, 2011). The amplitude of the proestrous LH surge in mice is greater than the estradiol-induced surge, attributable at least in part to augmented pituitary response to GnRH on proestrus (Silveira et al., 2017). In women, progesterone administration during the late follicular phase augments LH pulse amplitude, which could be attributable to increased amplitude GnRH release and/or increased responsiveness to endogenous GnRH, but does not alter pulse frequency, which would require central action (Hutchens et al., 2016). Of interest to the site of progesterone action, the excitability parameters observed in the present study on the afternoon of proestrus, when both progesterone and estradiol from the ovary were present before brain slice preparation, are remarkably similar to those during positive feedback in OVX+E daily surge mice (Adams et al., 2018), which have been exposed to only circulating estradiol for at least 2 d. These observations may indicate boosting effects of progestins on LH surge amplitude occur independent of GnRH neurons at the level of the anterior pituitary; such action could be directly on the pituitary and/or indirectly via alterations of other neuroendocrine factors that affect LH release such as gonadotropin-inhibitory hormone (Son et al., 2012).

In addition to the parameters examined in the present study, it is likely that other synaptic and intrinsic properties of GnRH neurons change with cycle stage. With regard to the former, estradiol suppresses glutamatergic excitatory fast synaptic transmission during negative feedback in the daily surge model (Christian et al., 2009), and increased glutamatergic transmission on proestrus in rats (Tada et al., 2013). In another estrogen-induced surge model, the density of spines, often considered a termination point for glutamatergic inputs, was increased in GnRH neurons expressing cFos as a marker of elevated neuronal activity during the surge (Chan et al., 2011). With regard to intrinsic properties, a decrease in both transient A-type and sustained delayed rectifier potassium currents (DeFazio and Moenter, 2002; Pielecka-Fortuna et al., 2011) and an increase in both low and high-voltage activated calcium currents have been reported during positive feedback using different estradiol regimens (Zhang et al., 2009; Sun et al., 2010). Similar changes in specific voltage-gated ion channels may underlie the changes in excitability observed among cycle stages in the present study. Of note, the lower excitability of cells recorded on the morning of proestrus and lower Rin compared to either the afternoon of diestrus or negative feedback (OVX+E AM) in the daily surge model may suggest greater changes in these and perhaps other conductances occur during the morning of proestrus (Adams et al., 2018).

The concept that estradiol regulates synaptic properties of GnRH neurons to bring about the switch from negative to positive feedback were not supported in recent work using another LH-surge induction model in which OVX mice are treated with basal estradiol replacement then an additional estrogen injection to mimic the proestrous estradiol rise (Bronson and Vom Saal, 1979b; Bronson, 1981). No differences were observed in sPSC or mPSC frequency between negative feedback (OVX+basal E, slices made 4.5–5 h before lights out, recordings made 1–3.5 h before lights off) and positive feedback (OVX+basal E + E injection, slices made 1.5–2 h before lights out, recordings 1 h before to 1.5 h after lights out; Liu et al., 2017). Despite this difference, both models reliably produce an LH surge. This could indicate that changes in GABAergic PSC frequency may not be necessary for initiating positive feedback but may mark cotransmission of other substances such as kisspeptin (Lee et al., 2010; Piet et al., 2018). In this regard, knockout of estradiol receptor α from GABAergic neurons eliminates estradiol positive feedback (Cheong et al., 2015). Of note, this would remove ERα from a large percentage of kisspeptin neurons in the anteroventral periventricular region that use GABA as a co-transmitter; the lack of a surge may reflect reduced activation of these neurons (Cravo et al., 2011; Frazao et al., 2013). Another possibility is that the overlap of recording time relative to lights out in the former study precluded detection of a difference between negative and positive feedback. If time of day interacts with estradiol to generate the changes observed in synaptic transmission to GnRH neurons, as suggested by the present data comparing proestrous AM and PM and previous work in the daily surge model, it is possible that the switch to positive feedback levels of transmission had already occurred based on basal estradiol alone. Of note, the frequency of synaptic transmission in that study is higher in all groups that we have observed either in daily surge or cycling mice.

The LH surge is critical for ovulation, reproduction and the continuation of species. The present studies add to a literature that indicates multiple factors can influence the switch from negative to positive feedback, and further indicates that the mechanisms producing negative feedback are also changing throughout the cycle. Feedback stage-dependent shifts in both GnRH neuron intrinsic excitability and fast-synaptic inputs likely contribute to the increase in firing rate and GnRH release during positive feedback.

## Synthesis

Reviewing Editor: Jeffrey Blaustein, University of Massachusetts Decisions are customarily a result of the Reviewing Editor and the peer reviewers coming together and discussing their recommendations until a consensus is reached. When revisions are invited, a fact-based synthesis statement explaining their decision and outlining what is needed to prepare a revision will be listed below. The following reviewer(s) agreed to reveal their identity: Richard Piet, Yoshitaka Oka.

The authors need to be more careful in comparing their present results (obtained at a single time point on two different stages of the estrous cycle) with those of Adams et al 2018 and Christian and Moenter 2007, which were obtained in OVX+E mice at different time-points. Perhaps the authors could alter their manuscript in order to relate their findings in diestrus vs proestrus (PM) to findings in OVX vs OVX+E (PM) reported in Adams et al 2018 and in Christian and Moenter 2007.

The experiments performed here do not examine the same effects as previous studies have in the OVX+E daily surge model. Studies in the daily surge model have examined GABAergic synaptic transmission and GnRH neuron excitability in the morning or in the afternoon (referred to as “negative” and “positive” feedback) with constant levels of circulating E. Although these previous papers have reported E-dependence of the effects observed (Adams et al 2018, Christian and Moenter 2007), one might argue that those changes reflect diurnal changes rather that shifts in E feedback from negative to positive. This issue needs to be addressed in the manuscript.

In this manuscript, GABAergic transmission and GnRH neuron excitability are assessed at two different stages of the estrous cycle, characterized by low circulating E (diestrus; negative feedback) and by high circulating E (proestrus; positive feedback), but at a single time point (i.e. at the expected time of surge onset).

In order to conclude that their observations match those of previous studies in OVX+E mice, the authors would need to provide data from diestrous and proestrous mice killed and recorded in the morning as well, so that effects of circulating E (negative vs positive feedback) can be fully untangled from those of time of day, unless they have another way to untangle this confound.

The grey shading illustrating in figure 3 the range of data obtained by Adams et al 2018 should be removed, as that dataset was obtained at two different times of day under constant circulating E, and, thus, cannot be fairly compared to the data obtained here at a single time-point in animals with different levels of circulating steroid sex hormones.

The authors should note that Christian et al 2009 reported E-dependent changes in glutamatergic excitatory fast synaptic transmission in OVX+E mice. Chan et al 2011 found that circulating E in a surge model increases spine density in those GnRH neurons activated at the time of the surge. Finally, Tada et al 2013 documented an increase in spontaneous excitatory PSCs in rat GnRH neurons on proestrus.

The authors seem to imply that the diestrus is equivalent to OVX+E AM. If so, it more supporting evidence is needed.

The authors should discuss which intrinsic ion channels may be modulated by E2.

Other comments:

Page 1, lines 13-14: “GABAergic transmission... was higher frequency ...” please revise this sentence.

Page 5, line 124: “Current was sampled at 10 kHz and filtered at 10 kHz”. This may just be a typo, but the sampling rate is equal to the upper limit of the filter, when it should be {greater than or equal to} 2 x the filter.

Page 6, lines 125-126: internal solution recipe was given in “data acquisition” and doesn't need to be repeated here.

Page 6: how many events were included? It is stated that at least two 120 second recordings were included for each cell, but it would be interesting to indicate a range for the number of events included.

This is important for the event distributions shown in figure 1, which are assumed to reflect the distributions of all events pooled from all recordings in the two groups. As proestrous GnRH neurons have more frequent PSCs, a few recordings which had much higher rates of PSCs than the rest might artificially shift the distribution leftward and thus exaggerate the overall effect. It is suggested that the authors also provide an analysis of the mean +/- sem of distributions in individual cells.

Page 8, line 174: “GABAergic transmission is primary...” should read “primarily”

Page 8 and figure 2: the authors should state the n numbers in the text.

Line 181, activity-independent neurotransmitter release is also dependent upon release probability at individual release sites.

Page 8, line 193-194: please change “minimum current required to initiate spikes” to “rheobase”. Same in Figure 3C.

Lines 197-198 is a repeat of the statement made just above (lines 193-194).

L254-256; the sentence “Together these observations may indicate some boosting effects of progestins on LH surge amplitude occur downstream of the GnRH network at the level of the anterior pituitary.” does not make sense. Can it be .... occurring downstream of...?

Although the authors' focus is on the mechanism of regulation of synaptic and intrinsic properties of GnRH neurons that is dependent on estrogen, the authors also used a lot of space for discussing the role of progesterone (L236-256 and next paragraph). Unfortunately, however, the authors did not do experiments to test the involvement of progestins in the present experiment. The authors are encouraged to more on their novel findings in their manuscript and less on progesterone.

## Author Response

We thank the reviewers and editorial team for their constructive comments on this manuscript We have used these suggestions to improve this manuscript presentation, add additional experiments and include additional commentary as detailed in this response. Changes in the revised manuscript are marked with blue text.

Computational Neuroscience Model Code Accessibility Comments for Author (Required):

N/A

Significance Statement Comments for Author (Required):

OK

Comments on the Visual Abstract for Author (Required):

N/A

Synthesis Statement for Author (Required):

The reviewers were mostly satisfied with the revisions to the manuscript. However, there are a still few issues that need to be dealt with to improve the clarity of the manuscript:

Critique: Figure 1B: p&lt;0.063 should read p=0.063

Response: Actually, this is correct. The exact p value was 0.062822, which was a bit bulky to put over the data.

Critique: Line 171 and figure 1C: Is the Kruskal Wallis test appropriate for comparing the cumulative distributions of interevent intervals?

Response: We consulted with a biomathematician on this question. Typically, we use Kolmogorov-Smirnov tests for comparison of distributions, however this test is limited to two groups and we now have three to compare. The suggestion we received was that ANOVA (Kruskal Wallis as our data are not normally distributed) was more rigorous, but that we should check pair-wise with KS tests as well. We did this, and the same comparisons were found different, with the same or more significant p-values for the pair-wise tests than for the post hoc Dunn's test as would be expected from the gain of power as well as the tendency of KS to find distributions with large numbers of values to be different.

Critique: Lines 209-214: the description of these results is a bit awkward. In particular, “Cells from proestrous AM mice further fired fewer spikes...” is difficult to follow. Please revise.

Response: This section has been revised to read “Specifically, at current steps from 12-30 pA, cells recorded on proestrous PM fired more spikes than cells from either diestrous PM mice or proestrous AM mice (Figure 3B, two-way repeated-measures ANOVA/Fisher's LSD, p&lt;0.05). Differences were also observed between the two negative feedback stages examined; at current steps {greater than or equal to}20 pA, cells from proestrous AM mice fired fewer spikes than cells from diestrous PM mice.”

Critique: Lines 319-324: the authors may also consider referring to studies showing that (i) knocking out the gamma2 subunit of GABAa receptors in GnRH neurons, which results in a substantial knockdown of PSCs in these cells, has little effect on fertility and in fact does not seem to affect the amplitude of estardiol-induced LH surges (Lee et al 2010, Endocrinology) and that (ii) while optogenetic stimulation of neurons in the AVPV/PeN results in GABA and kisspeptin release onto GnRH neurons, ultimately, only the neuropeptide seems to have a measurable effect on LH secretion (Piet et al 2018, Journal of Neuroscience).

Response: We have added these references as requested.

Critique: Lines 325-328: “...elements of the daily surge persist in the OVX+basal estradiol model...” In this reviewer's opinion, this is difficult to argue: if elements of the daily surge are maintained in that model, then diurnal changes in GABAergic synaptic transmission (such as those reported by Christian and Moenter in 2007) should be detected in that model.

Response: We have edited this sentence to read “Another possibility is that the overlap of recording time relative to lights out in the former study precluded detection of a difference between negative and positive feedback”. We cannot exclude the possibility that the changes induced by estradiol/estrogens do not continue to occur once brain slices are prepared, thus both time of preparation and of recording need to be considered when interpreting these results.

Critique: The sentence in Lines 30-33 appears incomplete: Both GABAergic transmission to GnRH neurons and GnRH neuron excitability are decreased during stages the estrous cycle characterized by negative feedback by gonadal steroids, vs on the late afternoon of proestrus, when positive feedback and ovulation occur.

Response: We believe this is a complete sentence but have edited it slightly to increase clarity.
